# Sustainable Phenylalanine-Derived SAILs for Solubilization of Polycyclic Aromatic Hydrocarbons

**DOI:** 10.3390/molecules28104185

**Published:** 2023-05-19

**Authors:** Illia V. Kapitanov, Surya M. Sudheer, Toshikee Yadav, Kallol K. Ghosh, Nicholas Gathergood, Vijai K. Gupta, Yevgen Karpichev

**Affiliations:** 1 Department of Chemistry and Biotechnology, Tallinn University of Technology (TalTech), 12618 Tallinn, Estoniavijai.gupta@sruc.ac.uk (V.K.G.); 2 School of Studies in Chemistry, Pt. Ravishankar Shukla University, Raipur 92010, India; kallolkghosh@gmail.com; 3 School of Chemistry, College of Science, University of Lincoln, Lincoln LN6 7TS, UK; ngathergood@lincoln.ac.uk; 4 Biorefining and Advanced Materials Research Centre, SRUC, Parkgate, Dumfries DG1 3NE, UK

**Keywords:** surface-active ionic liquids (SAILs), enzymatic decomposition, biodegradability, sustainability, solubilization, polycyclic aromatic hydrocarbons (PAHs)

## Abstract

The solubilization capacity of a series of sustainable phenylalanine-derived surface-active ionic liquids (SAILs) was evaluated towards polycyclic aromatic hydrocarbons—naphthalene, anthracene and pyrene. The key physico-chemical parameters of the studied systems (critical micelle concentration, spectral properties, solubilization parameters) were determined, analyzed and compared with conventional cationic surfactant, CTABr. For all studied PAH solubilization capacity increases with extension of alkyl chain length of PyPheOC_n_ SAILs reaching the values comparable to CTABr for SAILs with n = 10–12. A remarkable advantage of the phenylalanine-derived SAILs PyPheOC_n_ and PyPheNHC_n_ is a possibility to cleave enzymatically ester and/or amide bonds under mild conditions, to separate polycyclic aromatic hydrocarbons in situ. A series of immobilized enzymes was tested to determine the most suitable candidates for tunable decomposition of SAILs. The decomposition pathway could be adjusted depending on the choice of the enzyme system, reaction conditions, and selection of SAILs type. The evaluated systems can provide selective cleavage of the ester and amide bond and help to choose the optimal decomposition method of SAILs for enzymatic recycling of SAILs transformation products or as a pretreatment towards biological mineralization. The concept of a possible practical application of studied systems for PAHs solubilization/separation was also discussed focusing on sustainability and a green chemistry approach.

## 1. Introduction

Ionic liquids (ILs) have been widely used in many industries [1,2,3] and are one of the core focuses of research over the past two decades [4,5]. ILs are proposed as more desirable than conventional volatile solvents in many physical and chemical processes, often referred as “green” solvents [6]. They can be of natural origin and be prepared by a “benign by design” approach [5,7]. Designing ILs that lead to a reduction in the losses of solvents as well as less damage to the environment is an important aspect in green chemistry [6]. Ionic liquids in general fulfil many of the 12 criteria as a green solvent related to the availability, price, recyclability, synthesis, toxicity, biodegradability, performance, stability, flammability, storage, and renewability [8]. Ionic liquids can offer a better alternative to volatile solvents, which has led to its massive use in industrial applications such as separation and purification, and as chemical catalysts, biorefinery concepts [3], extractions [1] and others [9,10,11,12]

Recently, research studies have revealed that some of these ILs demonstrate a significant toxicity level [13,14]. Though the toxicity evaluations of ILs have been extensively reported in the literature, biodegradation data are comparatively limited [15]. Biodegradation is considered as the cleanest ultimate fate for compounds in nature. Although ILs can be easily synthesized, often in only a few steps, it is important to ensure that they are fully mineralizable in case of their introduction into the environment. ILs’ persistence in nature, due to their high stability, would result in adverse environmental toxicity [16]. It can lead to problematic wastewater pollution upon release into the environment because ILs, which are highly water soluble and not consistently biodegradable in wastewater aeration tanks, can have varying degrees of biodegradability [17]. The toxicity of ILs is related to the sorption of a surfactant molecule to biological membranes, which is driven by the nonspecific hydrophobic interactions [18]. Such adsorption of surfactants disrupts the cellular membranes and results in acute or chronic effects in microbes. The hydrophobic/hydrophilic balance of the molecule and the cationic charge density ultimately results in antimicrobial activity [19].

The synthesis and investigation of ILs using natural structural elements is a prospective approach in designing new molecules [7,20,21,22,23,24]. The ILs based on the amino acids are considered as one of the green alternatives and have high demand in industrial applications, which lead to the further development of a benign by design approach for the elaboration of an L-phenylalanine ethyl ester platform for designing completely mineralizable ILs [23,25]. After all, biodegradation of a newly synthesized compound can be firstly screened by test methods recommended by OECD guidelines [26,27] and the development in analytical methods such as LC-MS and NMR make it easier to detect the degraded products with a limited sample volume.

In order to describe ILs as a greener solvent, one should show complete and rapid biotic/abiotic degradation in nature. It is worth noting that there is often very little correlation between (i) the rate of chemical and enzymatic hydrolysis and (ii) the rate of biodegradation [28,29]. The biodegradability of ILs depends on their molecular structure [30] and the length of the alkyl chain [7,31,32]. Some ILs are stable to a wide range of chemicals as well as to high temperature, which are not expected to be readily biodegradable [33]. Pyridinium, cholinium, and imidazolium cations are the most studied among IL cations [34,35,36]. Pyridinium ILs are, in general, biodegradable to a higher extent than imidazolium ILs [23,25]. Thus, in the work [25], it was reported that the combination of ionic head groups with readily biodegradable biomolecules did not necessarily lead to an increase in biodegradability or degradation of the compound. The imidazolium- and pyridinium-derived phenylalanine ethyl ester ILs were shown to be more biodegradable than the proline and choline derivatives [23]. Recent studies provided an insight into the biodegradability of the ethyl ester of cationic phenylanine-derived ILs [24]. The pyridinium derivatives are found to be the preferred greener IL based on synthesis, toxicity, and biodegradation considerations [7]. Among ILs, special interests are the compounds, which contain significant hydrophobic fragments in their structure and demonstrate remarkable adsorption onto surfaces and change their properties (so-called surface-active ionic liquids, SAILs) [32].

The extraction and separation of polycyclic aromatic hydrocarbons (PAHs) are challenging problems [4] having both technological and environmental impacts [37]. Applying a water/surfactant system instead of organic solvents can particularly solve the aforementioned problems and make systems “greener” [38]. Water solutions of low toxic and biodegradable SAILs are prospective alternatives to conventional surfactants in the creation of sustainable ecologically friendly mixed compositions [39].

**Figure 1:**
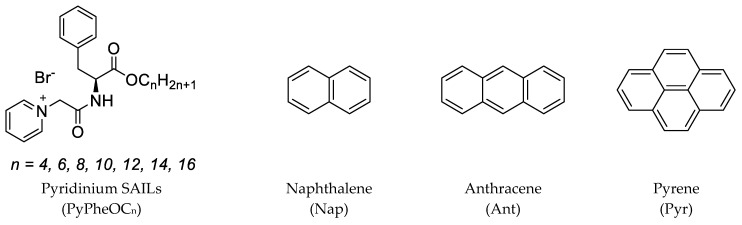
Structures of studied pyridinium SAILs and polycyclic aromatic hydrocarbons.

In this study, we evaluated the efficacy of pyridinium SAILs in the solubilization of model representative PAHs (naphthalene, anthracene and pyrene) in water/SAIL systems (Figure 1) and proved a strategy for direct degradation/hydrolysis of these SAILs by commercial enzymes. This concept can be applied in the design of recyclable environmentally friendly systems for PAHs solubilization/separation.

## 2. Results and Discussion

### 2.1. Study of Solubilization Capacity

The solubilization capacity of PyPheOC_n_ surfactants (n = 4, 6, 8, 10, 12, 14, 16) was evaluated, related to model representative PAH (naphthalene, anthracene and pyrene) and compared with the solubilization capacity of the widely used conventional cationic surfactant cetyltrimethylammonium bromide (CTABr) (Table 1).

**Figure 2:**
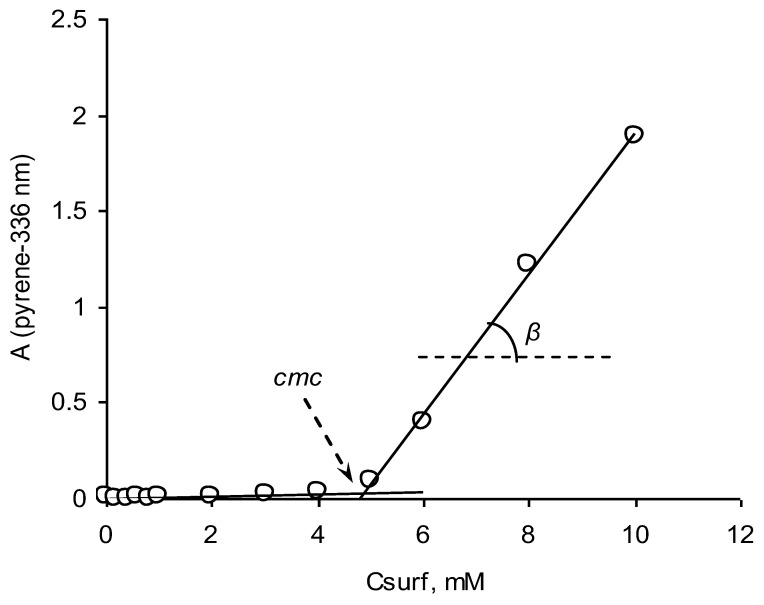
Dependence of the PAH absorbance vs. concentration of SAILs for the system PyPheOC_10_/pyrene: determination of *cmc* and *β* parameters.

The observed regularities for dependencies “concentration of PyPheOC_n_–PAH absorbance” are typical for such types of systems [38,40,41,42]: below the *cmc*, no dramatical changes of PAH concentration (absorbance) in the solution appeared, but beyond the *cmc*, an increasing of PAH concentration (absorbance) in the solution was observed (Figure 2). The brake-point on dependencies “concentration of PyPheOC_n_–PAH absorbance” corresponds to the *cmc* of the studied SAILs. The *cmc* values for PyPheOC_n_ SAILs and CTABr, obtained with different PAH, correspond well with each other and with the *cmc* of these compounds, determined using surface tension and conductivity measurements (Table 1; see also [32]).

**Figure 3:**
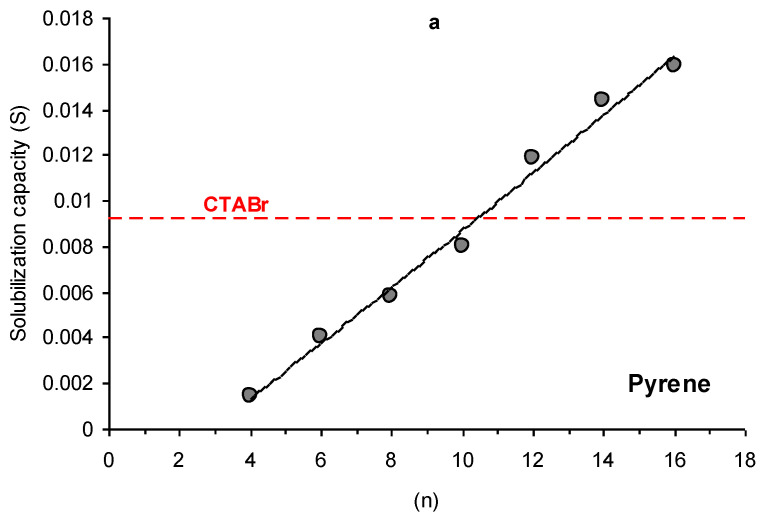

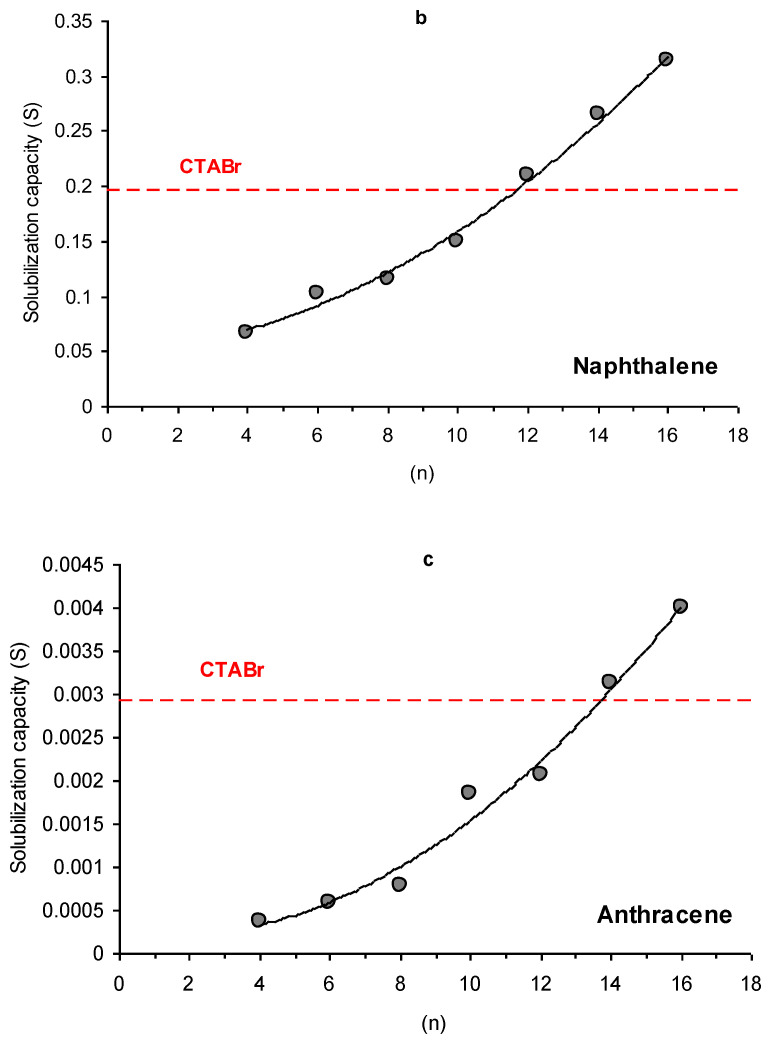
Dependencies of solubilization capacity (S) on alkyl chain length (n) of PyPheOC_n_ SAILs towards pyrene (**a**), naphthalene (**b**), and anthracene (**c**) compared to solubilization capacity of CTABr.

For all studied PAH, the solubilization capacity increases with an extension of the alkyl chain length of PyPheOC_n_ SAILs (Figure 3). The solubilization capacity, comparable with CTABr, demonstrates PyPheOC_n_ with n = 10–12 (naphthalene, pyrene) and n = 12–14 (anthracene). It corresponds to aggregation properties of the studied compounds: the *cmc* of CTABr are similar to PyPheOC_n_ with n = 10–12 (Table 1).

### 2.2. Study of Enzymatic Degradation/Hydrolysis of PyPheOC_n_ SAILs

Selecting a suitable enzyme which has higher degradation potential over the chemical compounds is very important [43,44]. Amidase and protease are the most representative groups of enzymes belonging to the class hydrolases in the remediation of polluted environments. The breakdown of ester, amide and peptide bonds by esterases, amidases and proteases may lead to products with little or no toxicity [45]. A previous report by Neumann et al. [46] shows that primary biodegradation of the test samples cyanomethyl side chain and its transformation product was enabled by a microorganism. This suggests that hydrolysis happened via nitrile degrading enzymes such as nitrilases or nitrile hydratases together with amidase. These studies support the role of the amidase enzyme as a catalyst for the biodegradation. Similar studies also reported that enzymes such as nitrilases and amidase are commonly applied as catalysts in organic synthesis for the hydrolysis of nitrile groups in the pharmaceutical industry and for bioremediation purposes, amongst others [47,48]. Protease is one of the most important groups of industrial enzymes, having a unique catalytic mechanism, broad substrate specificity and high robustness, which has widened their application into bioremediation and waste management [49].

This study again reveals the use of direct enzymes to accelerate the process of degradation in the wastewater treatment plants before the release of ILs to the environment so that we can reduce the chances of adverse toxicity effects in the future. It will be a promising finding and provide insightful information for the chemical industries, where they use a specific class of ILs that do not have any alternative replacements and are more persistent in the environment. Once we have the information on the degradability data as described in the current study, it is more applicable to change the molecular structure of the ILs to synthesize 100% biodegradable compounds. This is because chemical modification of the IL side chains may compromise the ability of the enzymes to recognize the modified structure as a suitable substrate and effect the complete degradation of the compound [50].

The commercial amidase enzymes purchased were obtained from the source *Escherichia coli* and *Achromobacter* with a penicillin hydrolytic activity unit of NLT 850 U/g and NLT 250 U/g, respectively. Protease enzymes were obtained from bacteria, fungi and plant sources with different enzyme activity as given in Table 2. Amidase enzymes are a large group of hydrolytic enzymes that contain a conserved stretch of approximately 130 amino acids. They are widespread, being found in both prokaryotes and eukaryotes. Amidase enzymes catalyze the hydrolysis of amide bonds, although they have a wide range of substrate specificity and function. Nonetheless, these enzymes maintain a core alpha/beta/alpha structure, where the topologies of the *N*- and *C*-terminal halves are similar. These enzymes possess a unique, highly conserved Ser-Ser-Lys catalytic triad used for amide hydrolysis, although the catalytic mechanism for acyl-enzyme intermediate formation can differ between enzymes [51], whereas protease enzymes work by hydrolyzing the peptide bonds and have the highest market share among industrial enzymes [52]. Proteases are classified as acidic, alkaline and neutral proteases according to the pH, and they exhibit maximum efficacy within a specific pH range. Proteases (also known as proteinases or peptidases) hydrolyze the peptide bond between amino acid residues in a polypeptide chain. Proteases may be specific and limited to one or more sites within a protein, or they may be nonspecific, digesting proteins into individual amino acids. Proteases are found in all organisms and are involved in all areas of metabolism [53].

In the present study, we revealed the enzymatic hydrolysis of pyridinium-based ILs of PyPheOC_4_ (as a representative example of SAILs of PyPheOC_n_ series), which have ester and amide bonds with amidase and protease enzymes. During the enzymatic hydrolysis, the enzymes enhanced the bond cleavage in molecules with water.

In theory, hydrolysis of an amide breaks the carbon–nitrogen bond and produces an acid and either ammonia or an amine. Though this reaction bears a resemblance to the hydrolysis of esters, there are, however, important differences. The hydrolysis of esters occurs relatively easily, whereas amides are much more resistant to hydrolysis. In the case of ester bonds, carboxylic esters readily hydrolyze to the parent carboxylic acid and an alcohol. Ester bond hydrolysis is the most prevalent type of biodegradation because they are a hydrolytically unstable functional group. Amides can be hydrolyzed only by heating for hours with a strong acid or strong base unless an enzyme is used. If amide hydrolysis occurs in a basic solution, the salt of the carboxylic acid forms, i.e., one mole of the base is required per mole of amide. If hydrolysis proceeds under acidic conditions, the ammonium salt of the amine is formed, and one mole of acid is required per mole of amide [54].

**Figure 4:**
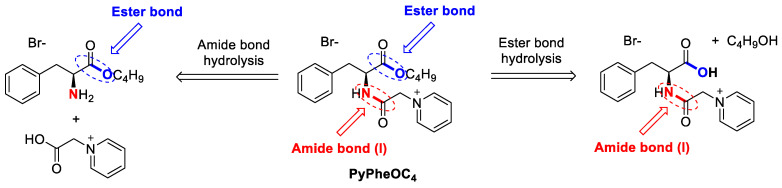
Expected pathways of C-O and C-N bonds hydrolysis in PyPheOC_4_.

The possible way by which ester bond and amide bond hydrolysis proceeded in the PyPheOC_4_ compound is illustrated in Figure 4.

When 2% PyPheOC_4_ was treated with 0.2 g amidase enzymes (1 and 2) at the incubation temperature of 40 °C, pH 5.5, shaking speed of 170 rpm, and 3 days incubation time, showed 55 to 80% ester bond hydrolysis. With amidase enzyme 1, 80% of the ester bonds were hydrolyzed whereas, with amidase 2, only 55% ester bond hydrolysis was observed. In both cases, no significant (less than 5%) amide bond hydrolysis product was recorded. The high stability of amide bonds is subjected to its propensity to form a resonating structure, which brings a double bond character to the amide CO-N bond [54].

When the concentration of PyPheOC_4_ was reduced to 1% (*w*/*v*) while maintaining the same incubation conditions as mentioned before with both amidase (1 and 2), 95% of the ester bond was hydrolyzed in PyPheOC_4_ and no significant amounts of amide bond break occurred. These studies revealed that with PyPheOC_4_, it was easier to hydrolyze the ester bonds by amidase enzymes compared to amide bonds. Moreover, the reduced concentration further made it feasible for the amidase enzymes to open more active sites for ester bonds. The transformation products formed from the studies are given in Figure 4. Based on the results, more enzymes were applied in this study in order to find complete hydrolysis of the compounds. Experiments were further carried out with 0.1 g of 1–20 types of protease enzymes (Table 2) along with amidase 1 and 2.

Based on the results mentioned above, the concentration of PyPheOC_4_ was maintained as 1% followed by an incubation temperature of 50 °C, shaking speed of 100 rpm and incubation for 7 days.

**Figure 5:**
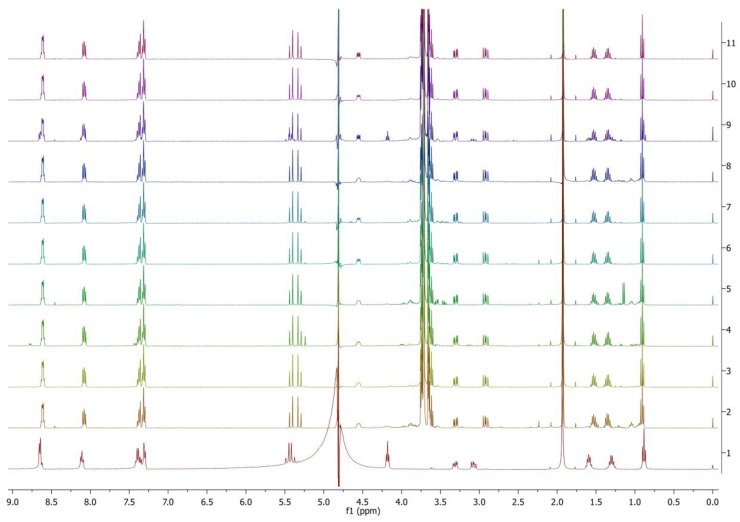
^1^H NMR spectra of PyPheOC_4_ solutions after incubations with selected protease enzymes (concentration of PyPheOC_4_ 1%). Spectrum 1 (bottom) corresponds to PyPheOC_4_ after incubation in buffer without enzymes; the spectra 2→11 represent incubation with enzymes P11→P20.

The applied protease enzymes work better at slightly higher temperatures, therefore the temperature 50 °C was selected for these test trials. The shaking speed was reduced to 100 rpm due to the optimum shaking speed recommended for the enzymes with an extended incubation time to 7 days. Among the 20 different types of protease enzymes tested on PyPheOC_4_, 100% ester bond hydrolysis was observed for all the samples except enzymes P8, P9 (86%) and P18 (79%); P13 also gives ca. 8% amidolysis; and representative examples of the NMR spectra are given in Figure 5.

Based on these results, we further reduced the concentration of the tested IL PyPheOC_4_ to 0.5% in order to study the possibility of complete hydrolysis of ester and amide bonds (100%). The optimum activity of the protease enzyme was reported with a pH of 6–7 [55]. Therefore, the pH of the sodium acetate buffer was further increased to 6.5 to improve the degradation rate. Incubation temperature, shaking speed and time were maintained at 50 °C, 100 rpm and 7 days, respectively.

**Figure 6:**
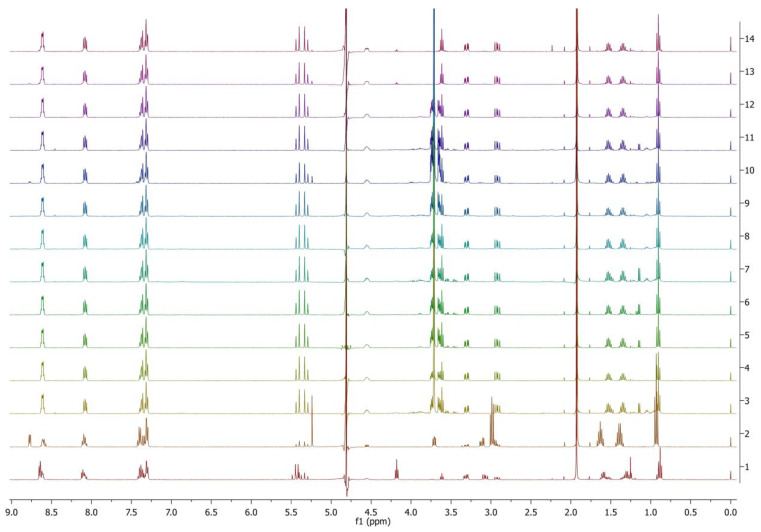
^1^H NMR spectra of PyPheOC_4_ solutions after incubations with selected protease enzymes at pH 6.5 (concentration of PyPheOC_4_ 0.5%). Spectrum 1 (bottom) corresponds to PyPheOC_4_ after incubation in buffer without enzymes; spectrum 2 recorded the model mixture of expected hydrolytic products; spectra 3–12 represent incubation with proteases P1, P2, P3, P4, P6, P10, P11, P13, P14, P17; spectra 13 and 14 represent incubation with amidase 1 and amidase 2, correspondingly.

Only selected protease enzymes from 1–20 that have high enzyme activity (P1, P2, P3, P4, P6, P10, P11, P13, P14, P17) and amidase 1 and amidase 2 were selected based on the previous test results. When the selected protease enzymes were used (based on higher enzyme activity), all the samples showed 100% ester bond hydrolysis with PyPheOC_4_. Among those, only P13 enzymes showed about 10–20% amide bond hydrolysis (Figure 6). In the case of amidase 1 and 2, only ester bond hydrolysis (100%) was observed.

**Figure 7:**
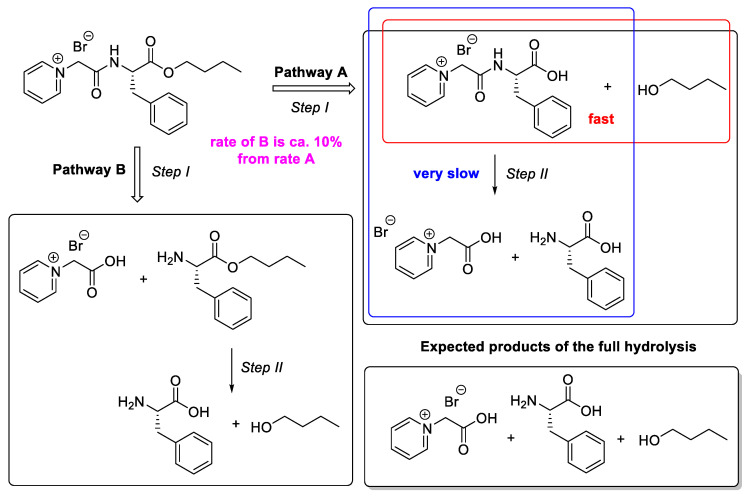
Degradation pathways analysis for PyPheOC_4_ based on NMR data.

PyPheOC_4_ in presence of the P13 enzyme showed a degradation pathway as presented in pathway A (90% of transformations) and pathway B (10% of transformations) (Figure 7). Transformations by pathway A are faster up to the first step (i.e., ester hydrolysis), whereas further hydrolysis of amide bonds in degradation product Py^(+)^-CH_2_-CO-NH-Phe-COOH is a very slow process. It can be connected to the reason that during the degradation process stabilizers, activators, or inhibitory products can form in the medium. which result from the material degradation or leaching out of enzyme additives, and those could affect the enzyme catalyzed reactions by influencing enzyme adsorption and activity, resulting from material degradation [56].

The intermediates and final products of enzymatic decomposition were not dependent on the degradation pathway (A or B) and were readily biodegradable compounds [24], which is critically important for the design of sustainable ecologically friendly systems.

### 2.3. Structural Modification of PyPheOC_4_ SAIL: Diamide Derivative PyPheNHC_4_

**Figure 8:**
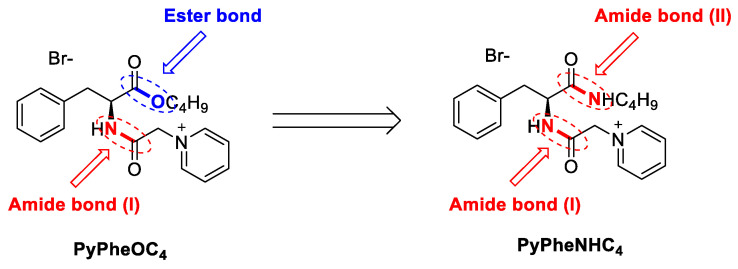
Structural modification of PyPheOC_4_ SAIL.

The change of ester bond in PyPheOC_4_ SAIL structure to amide bond can help to create compounds (Figure 8), which are more stable to alkaline hydrolysis [57,58] and can expand the possible application range of this type of compound, but also requires an evaluation of enzymatic decomposition.

The rate of enzymatic hydrolysis reaction is influenced by the physicochemical properties of the substrate and also by the inherent characteristics of a specific enzyme, which can be enzyme activity and its stability, local concentration, amino acid composition, and 3D conformation. Moreover, it is again very important to consider the medium conditions such as pH and temperature, since they strongly influence the properties of the substrate and the enzyme.

### 2.4. Study of Enzymatic Degradation/Hydrolysis of PyPheNHC_4_ SAIL

**Figure 9:**
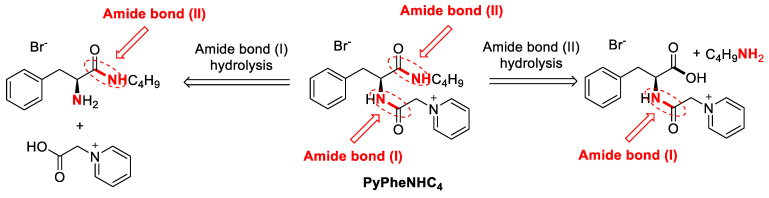
Expected ways of bonds hydrolysis in PyPheNHC_4_.

**Figure 10:**
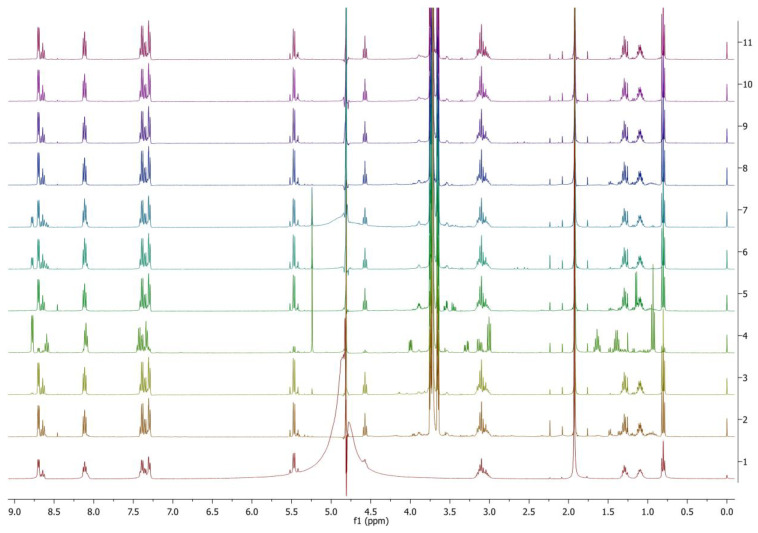
^1^H NMR spectra of PyPheNHC_4_ solutions after incubations with selected protease enzymes (concentration of PyPheNHC_4_ 1%). Spectrum 1 (bottom) corresponds to PyPheNHC_4_ after incubation in buffer without enzymes; the spectra 2→11 represent incubation with enzymes P11→P20.

It is expected that when compound PyPheNHC_4_ has been treated with enzymes, the hydrolysis may happen at either of these bonds, i.e., amide bond I or amide bond II, and ultimately lead to complete hydrolysis of the compound (Figure 9 and Figure 10).

The experimental trials and conditions explored and discussed before with PyPheOC_4_ have been tried with the new synthesized amide compounds. The first trial, i.e., 2% PyPheNHC_4_ with 0.2 g amidase enzymes (1 and 2) with an incubation temperature of 40 °C, pH of 5.5, shaking speed of 170 rpm, and 3 days incubation time, showed no evidence of amide bond hydrolysis with both the enzymes amidase 1 and 2. Thus, it is assumed that the stability of amide bonds is too strong to cleaved by amidase enzymes or is inaccessible to the active site. The high stability of amide bonds is subjected to its propensity to form a resonating structure, which brings a double bond character to the amide CO-N bond [54]. When the concentration of ionic liquids was reduced to 1% (*w*/*v*) by maintaining the same incubation conditions as mentioned before with both amidase enzymes (1 and 2) again, no traces of amide hydrolysis were observed. Experiments were further performed with 0.1 g of the 1–20 types of protease enzymes (Table 2) along with amidase 1 and 2. Among the 20 different types of protease enzymes tested with PyPheNHC_4_, the P1–P9 enzymes did not show any significant amide bond hydrolysis. Among the proteases P11–P20, the P13 enzyme showed 87% amide bond hydrolysis. The proteases P12, P15 and P16 demonstrated 10–20% amide bond hydrolysis (Figure 10). As shown in Table 2, all of the proteases were obtained from different sources. Among them, the P13 obtained from the source *Aspergillus oryzae* with an enzyme activity of 65 ELU/g worked well for hydrolysis of amide bonds in PyPheNHC_4_. It reveals that each enzyme has its optimum hydrolytic activity with the compounds under optimum conditions.

**Figure 11:**
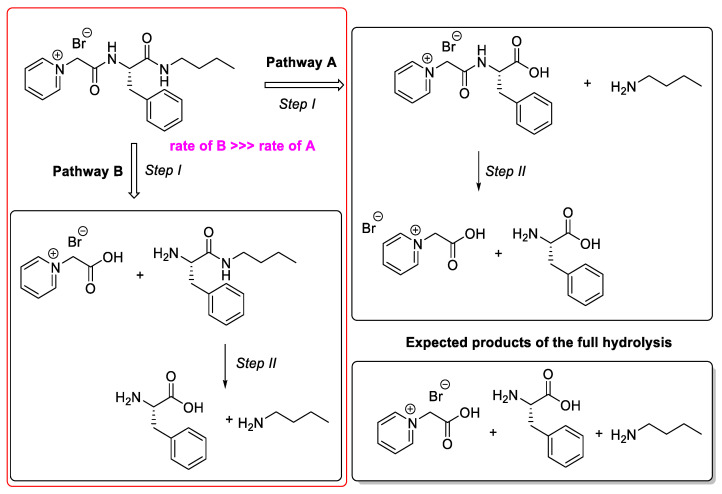
Degradation pathway analysis for PyPheNHC_4_ based on NMR data.

The pathway by which a protease enzyme breaks the amide bonds is illustrated in Figure 11.

**Figure 12:**
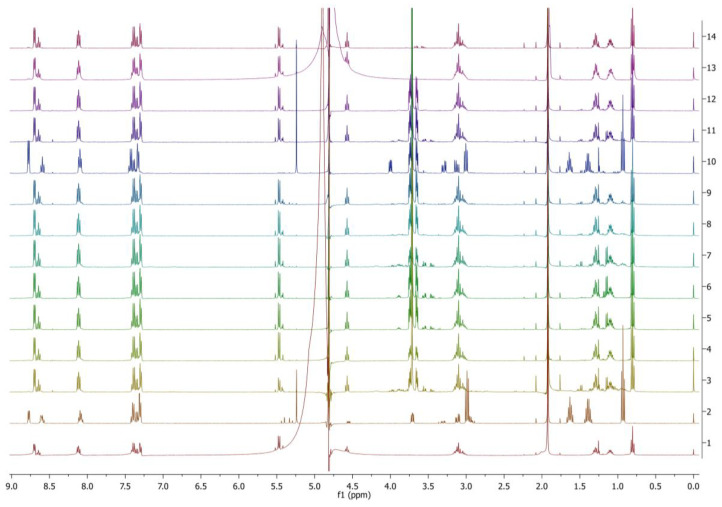
NMR spectra of PyPheNHC_4_ solutions after incubations with selected protease enzymes (concentration of PyPheNHC_4_ 0.5%). Spectrum 1 (bottom) corresponds to PyPheNHC4 after incubation in buffer without enzymes; spectrum 2 recorded the model mixture of expected hydrolytic products; spectra 3–12 represent incubation with proteases P1, P2, P3, P4, P6, P10, P11, P13, P14, P17; spectra 13 and 14 represent incubation with amidase 1 and amidase 2, correspondingly.

To enhance the complete degradation, the concentration of PyPheNHC_4_ was further reduced to 0.5% as investigated with PyPheOC_4_. The incubation temperature, shaking speed and time were maintained at 50 °C, 100 rpm and 7 days, respectively. Only selected protease enzymes from 1–20 that had high enzyme activity (P1, P2, P3, P4, P6, P10, P11, P13, P14, P17) and amidase 1 and 2 were selected based on the previous test results. With the reduced concentration (0.5%) of PyPheNHC_4_, the P13 enzyme showed 100% amide hydrolysis (Figure 12). PyPheNHC_4_ in the presence of the P13 enzyme showed 100% transformation by pathway B; i.e., hydrolysis of amide bond I. Transformations by pathway A by the hydrolysis of amide bond II is relatively slow in comparison with the rate of transformation by pathway B. The reaction showed a total cleavage of all amide bonds and observed Py^(+)^-CH_2_-COOH, phenylalanine and Bu-NH_2_ in the solution (Figure 11 and Figure 12).

### 2.5. Concept of PyPheOC_n_ and PyPheNHC_n_ SAILs Application to Solubilization of Polycyclic Aromatic Hydrocarbons

**Figure 13:**
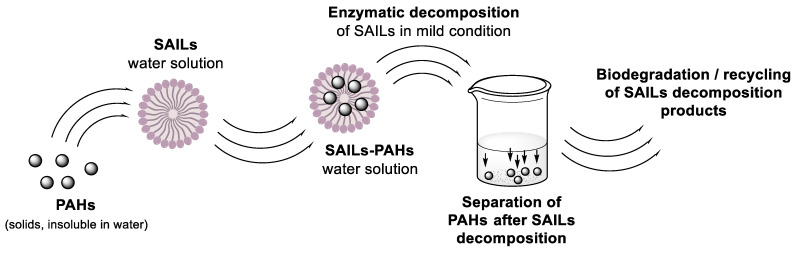
Concept of PyPheOC_n_ and PyPheNHC_n_ SAILs application to solubilization of PAHs.

The application of surfactants and SAILs in the design of effective and ecologically friendly sustainable systems for the solubilization of polycyclic aromatic hydrocarbons is very often limited due to the negative influence of surfactants and SAILs on the environment [14] and technological problems connected with the separation of solubilized PAH from surfactant solutions [4]. Among the SAILs PyPheOC_n_ and PyPheNHC_n_, which were the focus of our current study, there are several examples of compounds (with n = 4–8) that could be considered as low toxicity and readily biodegradable ILs [7]. An evaluation of the toxicity and biodegradability of potential hydrolytic decomposition products of PyPheOC_n_ and PyPheNHC_n_, performed in our recent studies [10,24,32], also confirm a great potential of these SAILs as a platform for ecologically friendly sustainable systems. The solubilization capacity of PyPheOC_n_ surfactants, evaluated related to model representative PAH (naphthalene, anthracene and pyrene), is comparable to the solubilization capacity of conventional cationic surfactant CTABr (see Table 1 and Figure 3; compare parameters for CTAB and SAILs with n = 8–12), but in the cases of PyPheOC_n_ and PyPheNHC_n_, polycyclic aromatic hydrocarbons could be easily separated from the SAILs water solution using enzymatic decomposition of SAILs under mild conditions. The decomposition of SAILs could be adjusted via the choice of enzyme system, reaction conditions and/or choice of SAILs type. Despite the possibility of solubilized aromatic carbons to have an impact on the enzymatic degradation of phenylalanine-derived SAILs, we suggest that enzymatic cleavage of surfactant monomers occurs [49]. Since the monomer is in the equilibrium with dynamic micellar aggregates [40], the drop in the monomer concentration will affect the micelle concentration in the system and thus reduce the PAH solubilized by the surfactant aggregates. The PAH initially bound by the micelles will be released in the bulk aqueous solution and precipitate. After SAILs decomposition, PAH, immobilized enzymes, and the water solution of SAILs decomposition products could be separated from each other using filtration techniques. The water solution of SAILs decomposition products could be completely biodegraded by the microorganisms in the environment or used for enzymatic re-synthesis of SAILs from decomposition products with future usage of the obtained solution for the next cycle of PAH extraction/solubilization (Figure 13).

## 3. Experimental Section

### 3.1. Materials

Naphthalene (Nap), anthracene (Ant) and pyrene (Pyr), cetyltrimethylammonium bromide (CTABr), inorganic and organic salts for preparation of working buffer solutions, acids and organic solvents were purchased from Sigma-Aldrich/Merck KGaA or Acros Organics/Fisher Scientific. Deuterated solvents for NMR analysis were purchased from Deutero GmbH (Kastellaun, Germany). Deionized water was prepared using the Direct-Q UV 5 water purification system.

Synthesis of SAILs PyPheOC_n_ is previously described [32]. Synthesis of PyPheNC_4_ was performed according to common synthetic procedures [10,24], confirmation of the structure and purity was performed using ^1^H, ^13^C, ^1^H-^1^H, ^1^H-^13^C, DETP 135 NMR techniques and by HRMS.

Amidase and protease enzymes (Table 2) were purchased from Fermenta Biotech Ltd. (Mumbai, India) and ChiralVision B.V. (Den Hoorn, The Netherlands), respectively. The enzymes were stored in a cold room until the start of the experiment.

### 3.2. Methods

#### 3.2.1. Study of Solubilization Capacity

Solubilization capacity of micellar systems was evaluated by determining the maximal solubility of the substrates (naphthalene, anthracene and pyrene) in surfactant solutions [38,40]. To a series of corresponding PyPheOC_n_ surfactant solutions (10–15 different concentrations in total, which cover concentration range before and after *cmc*; 3 mL of surfactant solution per each concentration) was added a fixed amount (5 mg per each concentration) of studied substrate, shaken intensively and leaved for equilibration for 48 h at 25 °C [38]. After equilibration, insoluble residue was filtered through Millipore filters (Durapore ^®^ PVDF membrane, pore size 0.22 µm), the filtrate was placed in the quartz cell and the UV-vis spectrum was recorded using UV-Vis spectrophotometer JASCO V-730 in the wavelength range from 200 to 400 nm. For studied solutions obtained value of absorbance (A) was recalculated on a pathlength 10 mm at the 311 nm (ε = 320 M^−1^⋅cm^−1^) for naphthalene, 378 nm (ε = 15,100 M^−1^⋅cm^−1^) for anthracene and 336 nm (ε = 62,800 M^−1^⋅cm^−1^) for pyrene. Molar extinction coefficients (ε, M^−1^⋅cm^−1^) for substrates were determined in independent experiments in hexane. Solubilization capacity of the surfactant (S) was calculated from the ratio S = β/ε, where β is the slope of the linear part of the A vs. C_surf_ dependence [38,41].

#### 3.2.2. Enzymatic Degradation/Hydrolysis Studies

In this experiment, two representative examples of pyridinium SAILs have been tested. Firstly, the experiments were started with compound PyPheOC_4_. Once the enzymatic degradability of this compound and its transformation products are obtained, the test trials were continued with the compound PyPheNHC_4_ (which is a result of structure improvement of PyPheOC_n_ SAILs series). Degradation tests were performed with different reaction conditions, such as doses of enzymes, pH, temperature, shaking speed and incubation period to optimize the best parameters for the test. We started the test trials with ester-based SAIL, PyPheOC_4_, with a concentration of 2% was prepared in 0.1 M sodium acetate buffer (pH 5.5). Then, 0.2 g of enzymes such as amidase 1 and 2 were weighed in separate 15 mL tubes. To this, 1 mL of the samples (i.e., ionic liquid in sodium acetate buffer) was added. The tubes were then placed in an incubator with a shaking speed of 170 rpm and a temperature of 40 °C for 7 days. Respective controls were kept without the addition of enzymes. The supernatants were then collected and stored in fresh tubes and preserved for NMR analysis; before recording the ^1^H NMR spectra, up to 20% *v*/*v* of D_2_O was added to a sample for locking.

After obtaining the results, similar tests were performed with a further concentration of PyPheOC_4_ (1% and 0.5%), doses of amidase 1 and 2, and protease 1–20 enzymes (0.1 g), pH (5.5, 6.5), temperature (50 °C), and shaking speed (100 rpm) (see Table 3). Once the test results were analyzed, similar trials were performed with the compound PyPheNHC_4_.

#### 3.2.3. NMR Analysis of Enzymatic Degradation/Hydrolysis Products

The NMR spectra were recorded on a Bruker Avance III 400 MHz spectrometer. For analysis, 400 µL of clear supernatant was used, which was transferred to NMR tubes and mixed with 100 µL of deuterium oxide (99.9%). NMR spectra were recorded using the standard water suppression method. Identification of enzymatic hydrolysis products were performed by comparison of ^1^H NMR spectra of potential transformation products with ^1^H NMR spectra of reaction mixtures of corresponding SAILs after incubation with the immobilized enzymes.

## 4. Conclusions

The solubilization capacity of a series of sustainable phenylalanine-derived surface-active ionic liquids (SAILs) was evaluated towards polycyclic aromatic hydrocarbons—naphthalene, anthracene and pyrene. The key physico-chemical parameters of the studied systems (critical micelle concentration, spectral properties, solubilization parameters) were determined, analyzed and compared with a conventional cationic surfactant, CTABr. For all studied PAHs, the solubilization capacity increases with an extension of the alkyl chain length of PyPheOC_n_ SAILs reaching the values comparable to CTABr for SAILs with n = 10–12. A remarkable advantage of the phenylalanine-derived SAILs PyPheOC_n_ and PyPheNHC_n_ consists in a possibility to cleave enzymatically ester and/or amide bonds under mild conditions, to separate polycyclic aromatic hydrocarbons in situ. A series of immobilized enzymes was tested to determine the most suitable candidates for tunable decomposition of SAILs. The decomposition pathway could be adjusted depending on the choice of enzyme system, reaction conditions and SAILs type. The evaluated systems can provide selective cleavage of the ester and/or amide bonds and help to choose optimal decomposition of SAILs for enzymatic recycling of SAILs transformation products or as a pretreatment towards biological mineralization. The concept of a possible practical application of the studied systems for PAHs solubilization/separation was also discussed, focusing on sustainability and green chemistry approaches.

## Figures and Tables

**Table 1:** Critical micelle concentration (*cmc*), *β* parameter and solubilization capacity (S) for PyPheOC_n_ surfactants, and cetyltrimethylammonium bromide (CTABr).

PyPheOC_n_ Chain Length (n)	*cmc*, mM					*β*			Solubilization Capacity (S)		
	Surface Tension	Conductivity	Solubilization								
			Nap	Ant	Pyr	Nap	Ant	Pyr	Nap	Ant	Pyr
4	57	89	80	90	80	21.4	5.6	90.4	0.067	0.00037	0.0014
6	11	19.9	25	23	26	32.7	9	251.9	0.102	0.00060	0.0040
8	2.25	4.9	5.2	3.7	4.9	36.9	11.7	366.6	0.115	0.00077	0.0058
10	0.65	1.6	1.6	1	1.4	48.1	27.9	503	0.150	0.00185	0.0080
12	0.19	0.5	0.2	0.1	0.3	67.1	31.3	745.7	0.210	0.00207	0.0119
14	0.056	0.18	0.1	0.07	0.08	84.9	47.4	905.2	0.265	0.00314	0.0144
16	0.0125	0.037	0.05	0.04	0.05	100.5	60.3	996	0.314	0.00399	0.0159
CTABr	0.9	1	1	1	0.8	62.5	43.9	573.6	0.195	0.00291	0.0091

Notes. Values of critical micelle concentration (*cmc*) for PyPheOCn surfactants, determinated by surface tension and conductivity methods, were taken from Kapitanov et al. [32]. Values of critical micelle concentration (*cmc*) for CTABr, determinated by surface tension and conductivity methods, were taken from Serdyuk et al. [38].

**Table 2:** Sources of enzymes used for enzymatic hydrolysis of studied ionic liquids.

Enzyme	Source	Activity
A1	*Escherichia coli*	NLT 850 U/g
A2	*Achomobacter*	NLT 250 U/g
E1	*Pichia sp.*	NLT 10,000 PLU/g
E2	*Pichia sp.*	NLT 10,000 PLU/g
P1	*Bacillus sp.*	400 ELU/g
P2	*Bacillus sp.*	750 ELU/g
P3	*Bacillus sp.*	100 ELU/g
P4	*Bacillus sp.*	275 ELU/g
P5	*Mucor miehei*	8 ELU/g
P6	*Bacillus licheniformis*	400 ELU/g
P7	*Bacillus amyloliquefaciens*	15 ELU/g
P8	*Geobacillus sp.*	5 ELU/g
P9	*Trichoderma reesei*	5 ELU/g
P10	*Bacillus subtilis*	225 ELU/g
P11	*Bacillus subtilis*	400 ELU/g
P12	*Aspergillus oryzae var.*	5 ELU/g
P13	*Aspergillus oryzae*	65 ELU/g
P14	*Bacillus subtilis*	150 ELU/g
P15	*Aspergillus niger*	5 ELU/g
P16	*Bacillus subtilis*	10 ELU/g
P17	*Bacillus subtilis*	175 ELU/g
P18	*Carica papaya*	5 ELU/g
P19	*Pineapple stem*	5 ELU/g
P20	*Fig tree latex*	5 ELU/g

**Table 3:** Experiment set up and reaction conditions of ILs enzymatic degradation studies.

Test	ILs Tested	IL Test Conc. (% *w*/*v*)	pH	Enzymes Tested	Enzyme Test Conc. (g)	Incubating Temperature (°C)	Incubation Speed (rpm)	Incubation Time (Days)
1	PyPheOC_4_ PyPheNHC_4_	2	5.5	Amidase 1 and 2	0.2	40	170	3 days
2	PyPheOC_4_ PyPheNHC_4_	1	5.5	Amidase 1 and 2	0.2	40	170	3 days
3	PyPheOC_4_ PyPheNHC_4_	1	5.5	Amidase 1 and 2 Protease 1–20	0.1	50	100	7 days
4	PyPheOC_4_ PyPheNHC_4_	0.5	6.5	Amidase 1 and 2 Protease (P1-P4, P6, P10, P11, P13, P14, P17)	0.1	50	100	7 days

## Data Availability

All the data gathered for this study are available in the article.

## Sample Availability

Samples of the compounds could be available from the authors upon request.
